# Deep Neural Network-Based Prediction of Temperature-Dependent Tensile Stress–Strain Behavior in Injection-Molded Impact-Modified Heat-Stabilized PA66 for Automotive Applications

**DOI:** 10.3390/polym18212629

**Published:** 2026-10-23

**Authors:** Román Eduardo Soto López, Iván Juan Carlos Pérez Olguín, Simón Yobanny Reyes López, Manuela Alejandra Zalapa Garibay

**Affiliations:** 1 Departamento de Ingeniería Industrial y Manufactura, Universidad Autónoma de Ciudad Juárez, Chihuahua C.P. 32320, Mexico; roman.soto@uacj.mx (R.E.S.L.); ivan.perez@uacj.mx (I.J.C.P.O.); manuela.zalapa@uacj.mx (M.A.Z.G.); 2 Laboratorio de Materiales Híbridos Nanoestructurados, Departamento de Ciencias Químico Biológicas, Instituto de Ciencias Biomédicas, Universidad Autónoma de Ciudad Juárez, Chihuahua C.P. 32310, Mexico; simon.reyes@uacj.mx

**Keywords:** polyamide 66, stress–strain prediction, temperature dependence, viscoelastic modeling, impact-modified nylon, heat stabilization, deep neural network

## Abstract

Deep neural networks (DNNs) have emerged as promising tools for predicting temperature-dependent tensile responses in engineering polymers. In this study, a temperature-dependent DNN-based predictive framework was developed to estimate the stress–strain response of impact-modified heat-stabilized polyamide 66 (PA66 IM HS) across a wide thermal range relevant to automotive applications. The proposed model was trained and validated using experimentally obtained tensile data collected between −40 °C and 150 °C. The developed framework successfully captured the nonlinear tensile stress–strain response observed experimentally across the investigated temperature range. Experimental results demonstrated a strong dependence of stiffness, tensile strength, ductility, and post-yield deformation on thermal conditions, with distinct behavioral transitions associated with the glass transition region and thermally activated deformation processes. Compared with conventional interpolation approaches, the proposed DNN model provided stable and physically coherent stress–strain predictions at experimentally characterized temperatures and by interpolation at intermediate temperatures within the investigated thermal domain. The model accurately reproduced elastic behavior, yielding transition, and post-yield deformation across the evaluated thermal range, achieving excellent agreement with the experimental curves. Additionally, a fracture-aware interpolation strategy was implemented to preserve experimentally observed rupture behavior at low temperatures. The obtained results demonstrate the applicability of the proposed DNN-based methodology as a computational tool for estimating temperature-dependent monotonic tensile stress–strain behavior within the investigated thermal and experimental domain.

## 1. Introduction

DNN-based predictive models offer several advantages over traditional constitutive equations in estimating the thermo-mechanical behavior of engineering materials. These advantages stem from the ability of DNNs to handle complex, non-linear relationships and large datasets, providing faster and more accurate predictions. DNNs can also incorporate data-driven insights and physics-based constraints, enhancing their applicability in diverse engineering scenarios [1,2].

Temperature-dependent viscoelastic and plastic deformation mechanisms significantly influence the mechanical performance of Polyamide 66 (PA66) in automotive engineering applications. These mechanisms affect the material’s stiffness, ductility, and overall durability, which are critical for components exposed to varying thermal environments, such as those found in automotive engines. The interplay between temperature, viscoelasticity, and plasticity determines how PA66 behaves under mechanical stress, impacting its suitability for specific applications [3,4,5]. Polyamides have been commercially available for nearly 80 years. The initial polyamide products were fibers derived from PA6 and PA66. The first PA66 fiber, known as Nylon, was launched in 1938, utilizing a production method established by W.H. Carothers at DuPont. Concurrently, PA6 fiber, referred to as Perlon, was introduced in Europe by IG Farbenindustrie, based on P. Schlack’s discovery regarding caprolactam polymerization [6].

PA66, also known as nylon 66, is a versatile engineering thermoplastic widely used in automotive components [7], fibers [8], films [9,10], and textile applications because of its excellent balance of mechanical performance, thermal resistance, processability, and relatively low production cost [11,12,13,14]. Its combination of strength and durability has made PA66 one of the most extensively employed polymers in demanding structural applications. PA66 exhibits high tensile strength, excellent fatigue resistance, and good dimensional stability, making it particularly suitable for components subjected to repetitive mechanical loading [15]. Moreover, its performance can be tailored through the incorporation of functional additives. For example, flame-retardant formulations can improve fire resistance while preserving mechanical integrity [16]. Modified magnesium hydroxide has also been shown to enhance both thermal stability and flame retardancy without significantly compromising the material’s mechanical properties [17]. These formulation strategies enable PA66 to meet the diverse performance requirements of engineering applications.

The stress–strain curve is one of the most important tools for evaluating the mechanical behavior of polymeric materials. It provides key mechanical parameters required to assess material performance [18], and allows the identification of elastic and plastic deformation regimes under applied loads, which is essential for predicting the structural reliability of engineering components subjected to different service conditions [19].

**Figure 1:**
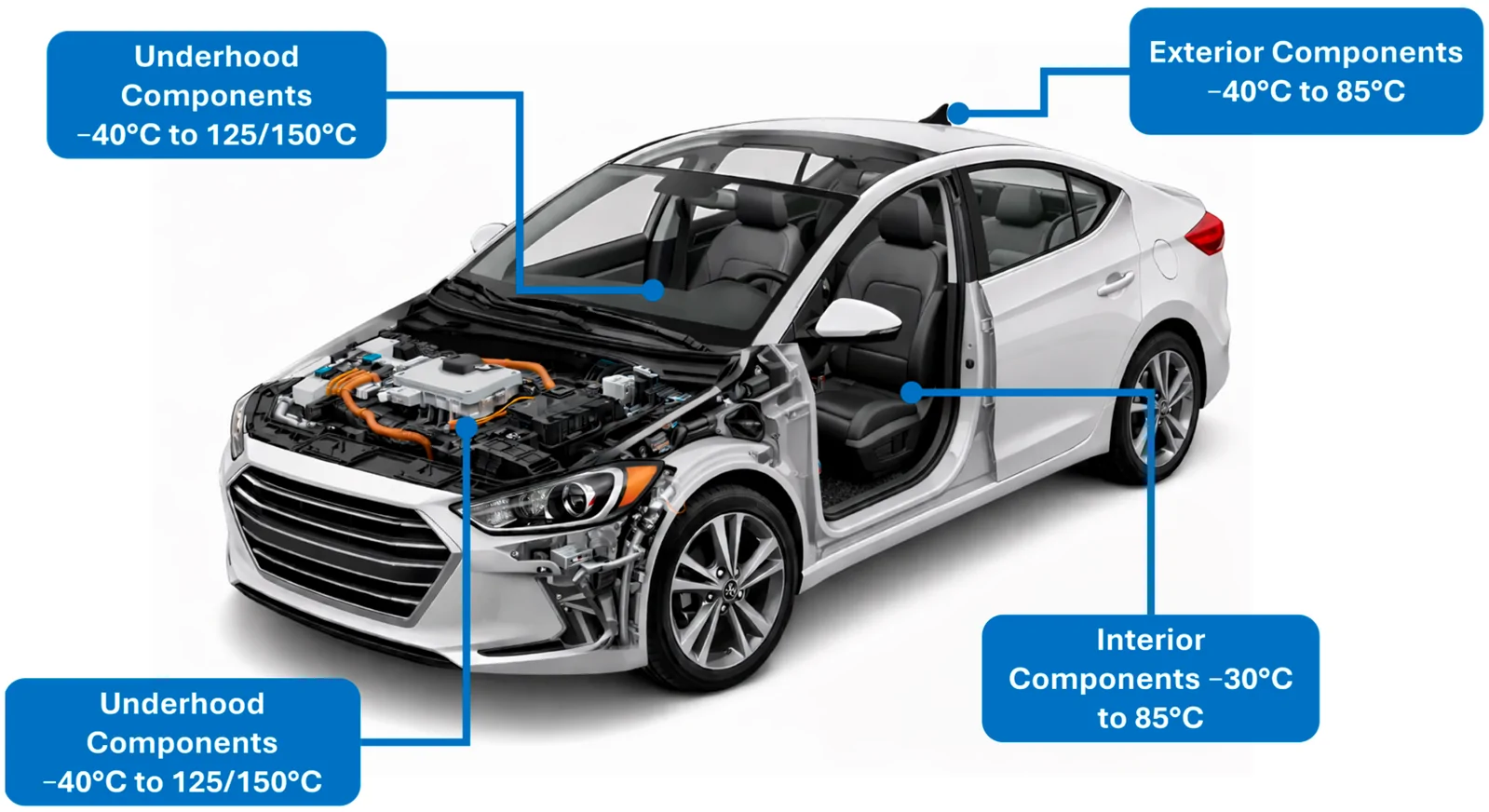
Representative thermal validation ranges for automotive components based on installation location within the vehicle.

From a design perspective, the stress–strain curve plays a crucial role in ensuring that materials operate within safe stress limits, particularly in industries such as automotive and aerospace, where failure can have severe consequences [20,21]. Additionally, the mechanical behavior of polymers such as PA66 is highly dependent on temperature, and the stress–strain curve provides valuable insight into how these properties evolve under different thermal conditions [22,23]. This is especially important in automotive environments, where components are exposed to temperature ranges from −40 °C to 85 °C or higher, depending on their location within the vehicle as shown in Figure 1.

The experimental temperatures were selected to cover a broad range of thermal conditions relevant to automotive components. In particular, ISO 16750-4:2023 defines climatic loads and operating-temperature ranges for electrical and electronic equipment according to vehicle mounting conditions. The standard includes maximum operating-temperature classifications of 85 °C, 105 °C, 125 °C, and 150 °C, all within classifications extending to a minimum operating temperature of −40 °C [24]. Accordingly, these temperatures were selected as representative reference levels for progressively elevated automotive thermal environments, rather than as temperatures corresponding exclusively to a single vehicle location. Together with the intermediate conditions at 0 °C and 23 °C, the experimental program therefore spans low-temperature, ambient, and progressively elevated thermal conditions relevant to automotive component characterization.

The stress–strain curve is also essential for failure analysis, as it allows distinguishing between brittle and ductile behavior, as well as understanding long-term phenomena such as fatigue and creep under cyclic or sustained loading conditions [25,26,27,28]. A lack of accurate stress–strain data can lead to critical engineering issues, including structural failures, poor material selection, unexpected deformation, and safety hazards in high-demand applications [29,30,31,32,33]. Moreover, insufficient understanding of these properties may result in inefficient designs, increased costs, and non-compliance with industry standards such as ASTM and ISO [34,35,36,37,38,39,40]. Several engineering failures reported in automotive and aerospace applications have demonstrated the importance of accurately understanding material behavior under combined mechanical and thermal loading conditions [41,42,43,44,45,46,47,48,49]. The stress–strain curve is essential for evaluating the shape memory effect, strain hardening, and viscoelastic behavior of plastics, which are critical for their application in engineering and industrial processes. Some examples of it are the shape memory effect which indicate the material’s ability to return to its original shape after deformation [50].

Recent data-driven approaches have demonstrated the capability of artificial neural networks to represent nonlinear stress–strain relationships under varying thermomechanical conditions. Depending on the intended application, these models may incorporate strain, temperature, strain rate, deformation history, or other state variables as network inputs. More comprehensive ANN-based constitutive frameworks have been developed using extensive experimental datasets and multiple loading conditions, allowing the effects of temperature, strain rate, and loading history to be incorporated into the predicted mechanical response. However, such approaches generally require substantially larger and more diverse datasets, while purely data-driven constitutive models may also face limitations associated with incomplete sampling of the experimental domain and the absence of explicit physical constraints [51,52].

For temperature-dependent tensile characterization of engineering polymers, an alternative approach is to formulate a reduced data-driven mapping within a controlled experimental domain. In this case, strain and temperature can be used as model inputs and stress as the output when strain rate, loading mode, and specimen conditioning are maintained under the prescribed experimental conditions. Such a formulation does not constitute a general thermo-viscoelastic constitutive law, but it can provide a practical means of representing and interpolating experimentally observed tensile responses across discrete thermal conditions. The reliability of such an approach depends strongly on the available temperature coverage, the number of experimental specimens, and the validation strategy used to assess interpolation between measured conditions.

Despite the increasing application of data-driven methods to constitutive and stress–strain modeling, comparatively limited attention has been given to reduced-input neural-network frameworks specifically designed to interpolate complete tensile stress–strain responses of impact-modified, heat-stabilized PA66 across a broad sub-zero-to-elevated temperature range using experimentally measured curves from multiple specimens at each thermal condition. In particular, the combination of experimentally characterized PA66 IM HS, a temperature range from −40 °C to 150 °C, multiple tensile specimens per temperature, and a fracture-aware constraint for low-temperature predictions provides a specific framework for evaluating temperature-dependent tensile interpolation while explicitly preserving the experimentally observed fracture domain.

The objective of this study is to develop and evaluate a reduced-input DNN framework for estimating the temperature-dependent tensile stress–strain response of injection-molded PA66 IM HS as a function of strain and temperature. The experimental investigation covers a temperature range from −40 °C to 150 °C, selected to represent thermal conditions relevant to automotive applications, including low-temperature environmental exposure and elevated under-hood operating conditions. The model was trained using experimentally obtained tensile curves from five individual specimens at each of seven discrete temperatures and was evaluated for its ability to interpolate stress–strain responses between experimentally characterized thermal conditions. A fracture-aware post-processing strategy was additionally incorporated in the low-temperature regime to constrain the predicted curves according to the experimentally observed fracture behavior. Unlike purely numerical simulations, the proposed methodology is based on experimental tensile data obtained from multiple physical specimens, allowing the model to represent the experimentally observed temperature-dependent mechanical trends and specimen-to-specimen variability. The proposed framework is specifically intended for interpolation of tensile behavior within the investigated temperature range and under the prescribed experimental conditions; it does not constitute a general thermo-mechanical constitutive model and does not account for strain-rate dependence, loading-history effects, moisture evolution, or multiaxial loading conditions.

## 2. Materials and Methods

### 2.1. Materials

The material employed in the present investigation was an impact-modified, heat-stabilized polyamide 66, designated as PA66 IM HS, which was chosen owing to its utilization in automotive components that experience fluctuations in thermal and mechanical conditions. This polymer is classified within the aliphatic polyamide category and possesses attributes that are advantageous for engineering applications, including mechanical robustness, thermal resilience, and favorable processability. Table 1 summarizes the key properties of PA66 IM HS.

To obtain the experimental data, tensile tests were performed on injection-molded PA66 IM HS specimens according to ASTM D638 using Type I specimens at a crosshead speed of 50 mm/min. The specimens were manufactured under industrial injection-molding conditions. Detailed processing parameters, including the screw temperature profile, mold temperature, injection speed, injection pressure, holding pressure and time, cooling time, and cycle time, cannot be disclosed due to industrial confidentiality restrictions. Information regarding specimen orientation relative to the injection-molding flow direction is subject to the same confidentiality restrictions. Consequently, the potential influence of processing history and specimen orientation on the measured tensile response could not be independently evaluated and is acknowledged as a limitation of the present study.

The specimens were evaluated under different thermal conditions to investigate the temperature-dependent mechanical response of the material. For each designated test temperature, five individual specimens were tested to capture the experimental variability of the material and improve the robustness of the predictive model. Detailed production-batch traceability of the injection-molded specimens cannot be disclosed due to industrial confidentiality restrictions. Therefore, the available experimental records do not allow the specimens to be classified as originating from the same or from independent molding batches. This limitation is explicitly acknowledged in the present study.

The dataset used for model development comprised experimental stress–strain curves obtained under different thermal conditions. Each curve included measurements of axial deformation, stress, and temperature, which were systematically compiled into a database for subsequent analysis and training of the predictive model. The parameters considered were axial deformation, mechanical stress, and testing temperature. The mechanical tests were performed under seven discrete thermal conditions selected to represent thermal operating environments relevant to automotive applications. The investigated temperatures were −40 °C, 0 °C, 23 °C, 85 °C, 105 °C, 125 °C, and 150 °C.

### 2.2. Experimental Characterization

The thermal characterization of the material was conducted using Differential Scanning Calorimetry (DSC) and simultaneous thermal analysis. DSC measurements were performed using a DSC Q200 (TA Instruments, New Castle, DE, USA) (Universal Analysis V4.5A) at a heating rate of 10 °C/min under a nitrogen atmosphere. The thermal program consisted of a first heating cycle to 200 °C, cooling to 25 °C, and a second heating cycle to 200 °C, using a sample mass of 10.06 mg. The DSC Q200 analysis was used to examine the thermal response during the first heating and cooling and second heating cycles. The present study did not include quantitative determination of crystallization enthalpy, melting enthalpy, or degree of crystallinity. Complementary simultaneous thermal analysis was performed using an SDT Q600 (TA Instruments, New Castle, DE, USA), with software version V20.9 Build 20, at a heating rate of 10 °C/min under an argon atmosphere over a temperature range from 26 °C to 600 °C. The heat-flow signal obtained from the SDT Q600 analysis was used to identify the glass-transition region and melting transition, while the thermogravimetric signal was used to assess the thermal stability and degradation characteristics of the material.

#### ATR-FTIR (Attenuated Total Reflectance Fourier-Transform Infrared) Characterization

ATR-FTIR analysis was performed as a complementary material-identification technique to verify that the chemical structure of the tested material was consistent with PA66 prior to mechanical characterization and DNN modeling. The analysis was not intended to quantify moisture content, crystallinity, hydrogen-bonding effects, or temperature-dependent molecular mechanisms.

**Figure 2:**
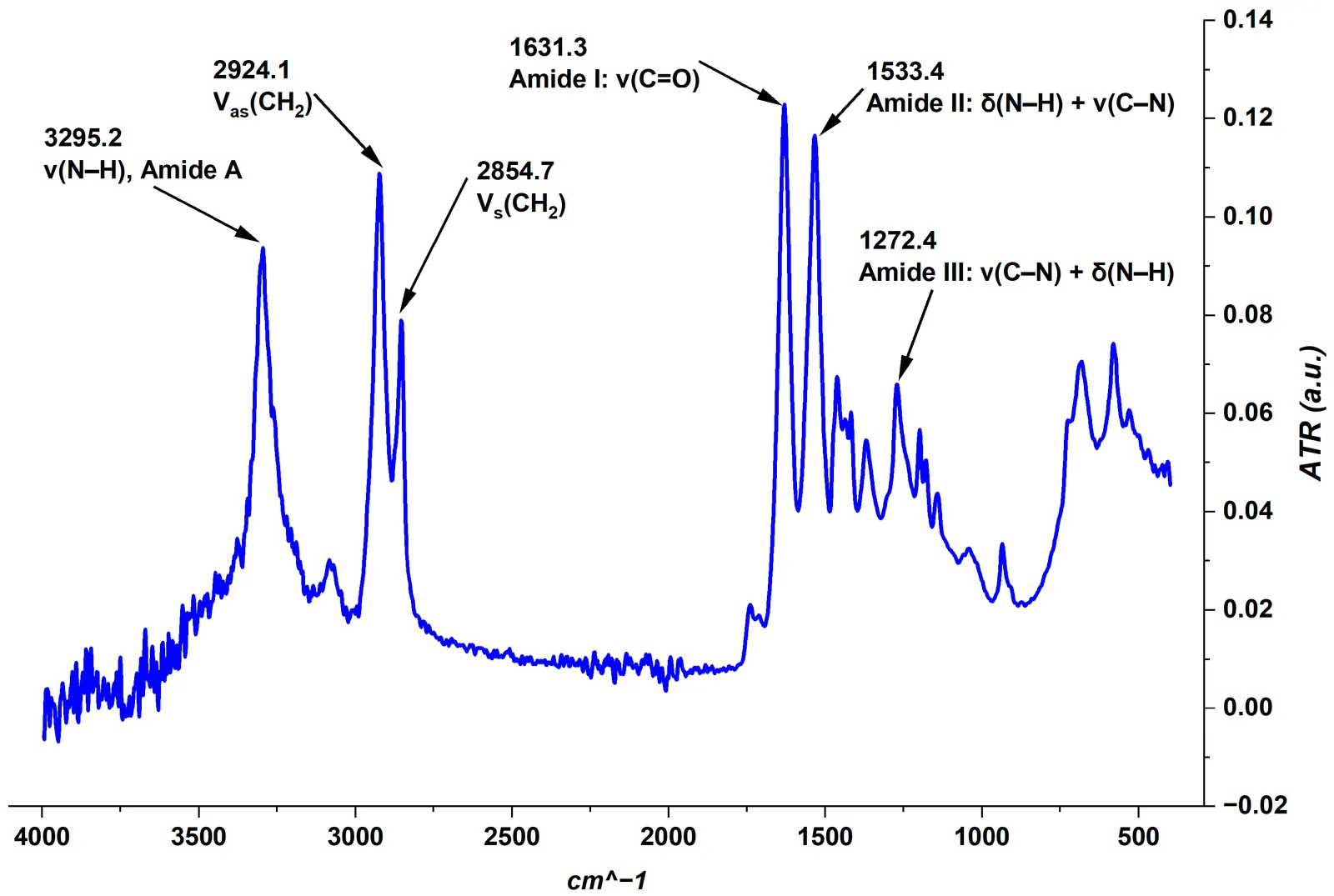
ATR-FTIR spectrum of PA66 IM HS showing characteristic amide (I, II, III, A) and CH_2_ vibrational bands consistent with the chemical structure of PA66.

The ATR-FTIR spectrum of PA66 IM HS showed the characteristic functional groups associated with polyamide 66. The absorption band at approximately 3295.2 cm^−1^ corresponds to N–H stretching (Amide A), while the peaks at 2924.1 and 2854.7 cm^−1^ are associated with asymmetric and symmetric CH_2_ stretching vibrations, respectively. Additionally, the characteristic amide bands observed at 1631.3 cm^−1^ (Amide I), 1533.5 cm^−1^ (Amide II), and 1272.4 cm^−1^ (Amide III) are consistent with the expected chemical structure of PA66, as shown in Figure 2.

### 2.3. Computational Environment

All computations were performed on a workstation equipped with a 13th Gen Intel(R) Core (TM) i7-13700HX processor and 32 GB of RAM, running Windows 11 on a 64-bit operating system, using MATLAB Version R2026a.

### 2.4. DNN Development

**Figure 3:**
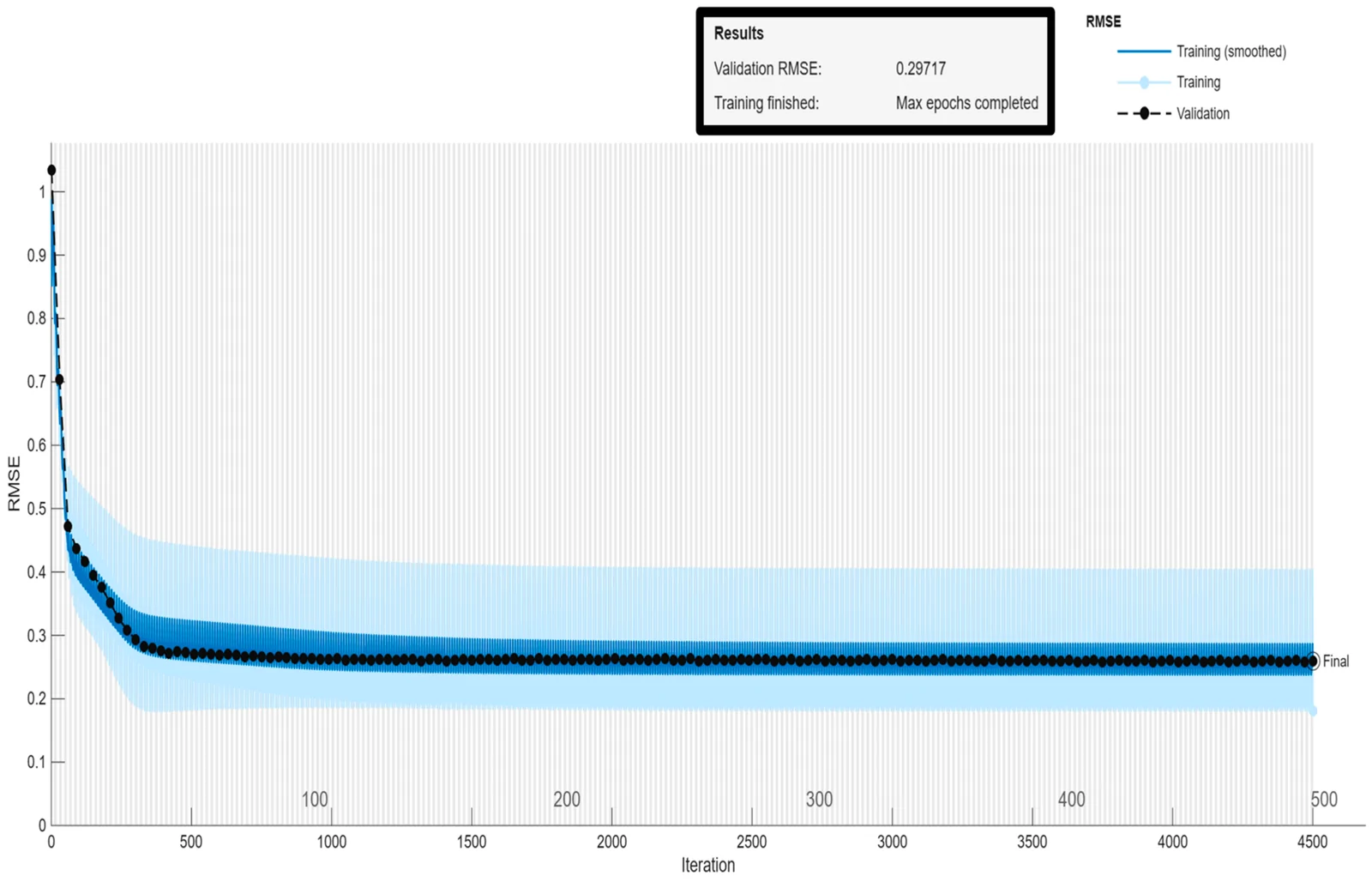
Training history of the proposed DNN showing the evolution of the root mean square error (RMSE). The solid blue line represents the smoothed training RMSE, the light-blue markers represent individual training RMSE values, and the black markers indicate validation RMSE. Due to the high density of training iterations, the light-blue markers overlap and are difficult to distinguish individually. The apparent background pattern results from the overlapping graphical elements and does not represent an additional dataset or uncertainty interval.

**Figure 4:**
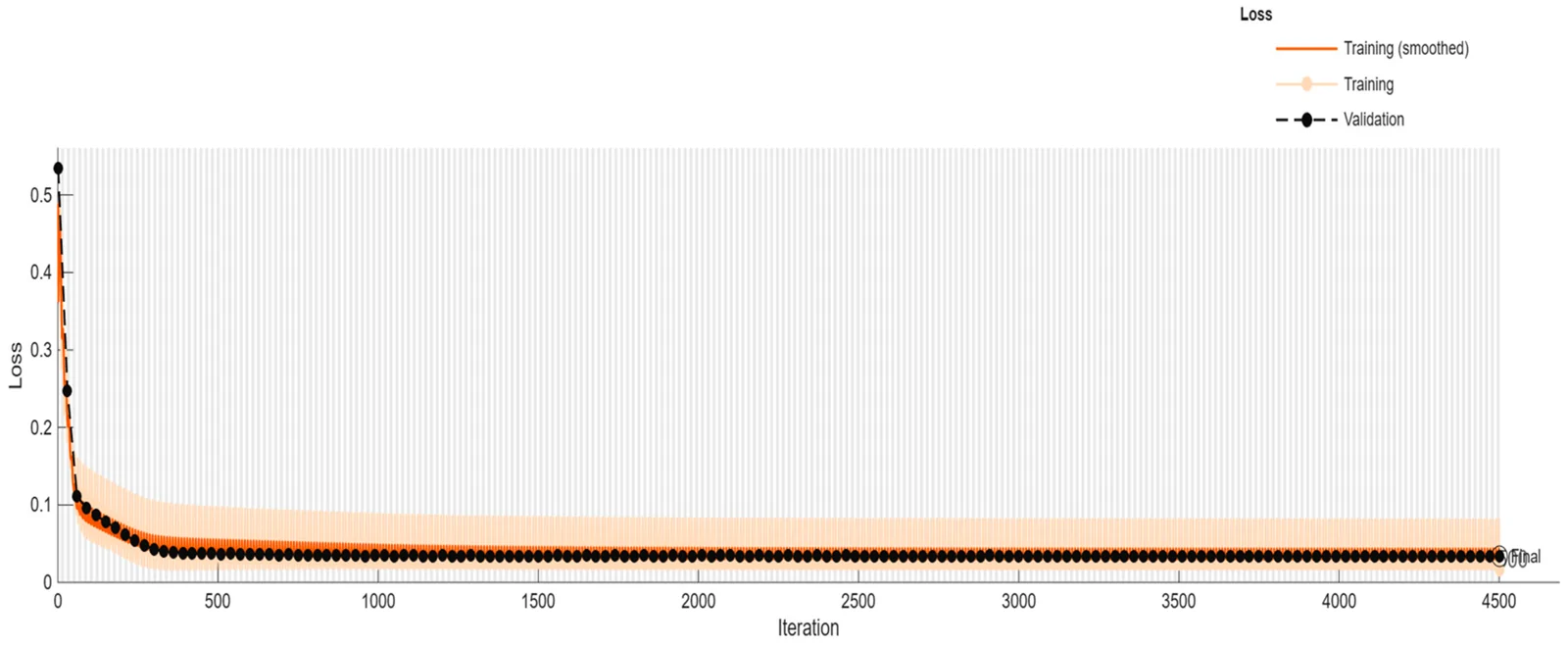
Training history of the proposed DNN showing the evolution of the loss function. The solid orange line represents the smoothed training loss, the light-orange markers represent individual training loss values, and the black markers indicate validation loss. Due to the high density of training iterations, the light-orange markers overlap and are difficult to distinguish individually. The apparent background pattern results from the overlapping graphical elements and does not represent an additional dataset or uncertainty interval.

The experimental dataset was initially stored in Microsoft Excel and subsequently imported into MATLAB R2026a for preprocessing, training, and validation of the predictive DNN model. The input variables consisted of strain and temperature, while stress was defined as the prediction target. Prior to training, all variables were normalized using standard score normalization to improve convergence stability. The evolution of the root mean square error (RMSE) and loss during model training and validation is presented in Figure 3 and Figure 4. The convergence of both metrics indicates stable model training with a final validation RMSE of 0.29717 after completion of the maximum number of epochs.

**Figure 5:**
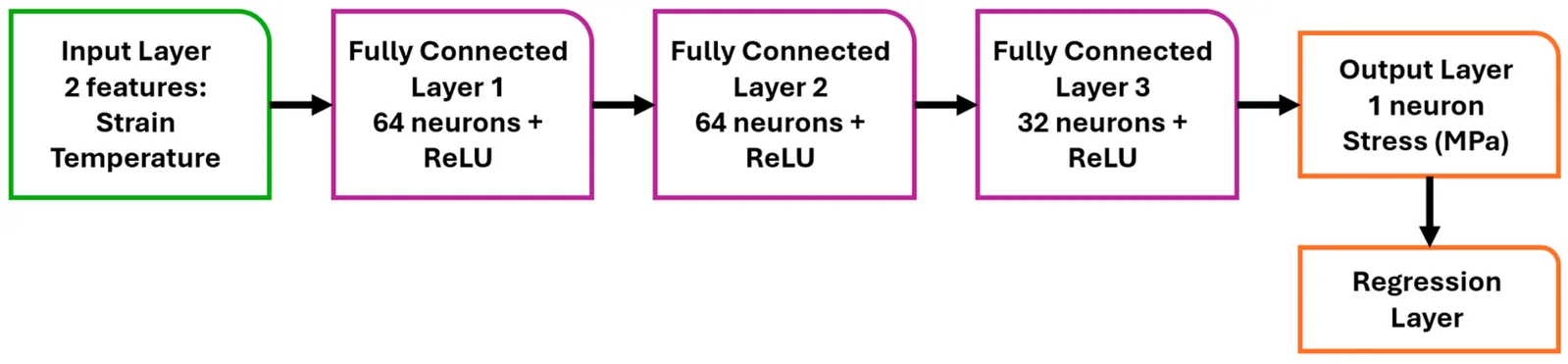
Architecture of the DNN model developed for temperature-dependent stress–strain prediction of PA66 IM HS using strain and temperature as input variables.

The deep neural network was trained using the Adam optimization algorithm with a maximum of 500 epochs, a mini-batch size of 128, and an initial learning rate of 0.001. The dataset was randomly divided into training, validation, and testing subsets. Three fully connected hidden layers containing 64, 64, and 32 neurons, respectively, were implemented using ReLU activation functions, followed by a regression output layer for stress prediction as shown in Figure 5.

The scope of the proposed DNN is limited to the prediction of temperature-dependent monotonic tensile stress–strain behavior under the experimental conditions investigated in this study. Strain and temperature were used as model inputs, while stress was defined as the prediction target. Therefore, strain-rate effects, moisture dependence, time-dependent behavior, and loading-history effects are not explicitly represented by the present model, and the proposed framework should not be interpreted as a general thermo-mechanical constitutive model.

#### 2.4.1. Leave-One-Temperature-Out Validation

To further evaluate the interpolation capability of the proposed DNN under temperature conditions not included during model development, a leave-one-temperature-out (LOTO) validation procedure was implemented. The five interior experimental temperatures (0 °C, 23 °C, 85 °C, 105 °C, and 125 °C) were individually excluded from the dataset, while the boundary temperatures of −40 °C and 150 °C were retained because their exclusion would require extrapolation beyond the remaining experimental temperature range. For each LOTO case, an independent DNN was retrained using the same network architecture and training hyperparameters employed in the original model. The normalization parameters were calculated exclusively from the remaining development data, ensuring that the withheld temperature did not contribute to model training, validation, testing, or data normalization. The complete experimental dataset corresponding to the excluded temperature, comprising five tensile specimens, was subsequently used as an external holdout condition to evaluate the predicted stress–strain response. Predictive performance was quantified using the root mean square error (RMSE), mean absolute error (MAE), and coefficient of determination (R^2^). To maintain independence between the withheld experimental condition and the prediction procedure, the fracture-aware post-processing strategy was not applied during the LOTO evaluation. A fixed random seed (rng(42, ‘twister’)) was used to ensure reproducibility across the five validation cases.

#### 2.4.2. Fracture Interpolation Strategy

To improve the physical consistency of the predictive model at low temperatures, a fracture-aware interpolation strategy was implemented for the temperature range between −40 °C and 23 °C. The temperatures of −40 °C, 0 °C, and 23 °C were selected because they represent the temperature region where fracture occurred within the experimentally measured strain range and where the material exhibited a more brittle response. Temperatures above 23 °C showed substantially higher ductility and fracture strains beyond the strain range required for the proposed interpolation framework. Therefore, the fracture-aware interpolation strategy was focused on the low-temperature regime where premature fracture could lead to non-physical stress predictions. The fracture point of each experimental stress–strain curve was identified based on the abrupt stress drop occurring after the maximum stress region. The corresponding fracture strain values at −40 °C, 0 °C, and 23 °C were then used to estimate the expected fracture strain at intermediate temperatures by linear interpolation. A linear interpolation approach was adopted because the experimentally measured fracture strains exhibited a monotonic and approximately linear increase within this restricted temperature interval, while the limited number of available data points did not justify the use of higher-order fitting functions. During prediction, the generated stress–strain curve was automatically truncated once the interpolated fracture strain was reached, preventing the model from producing non-physical stress values beyond the experimentally observed deformation limit. This strategy allowed the proposed DNN model to preserve experimentally observed rupture behavior while maintaining continuous temperature-dependent interpolation capability within the investigated thermal range.

### 2.5. AI-Assisted Figure Generation

The illustrative vehicle image presented in Figure 1 was generated using ChatGPT (OpenAI, GPT-4o). The AI-generated content was limited exclusively to the vehicle illustration. The temperature ranges and corresponding annotations were independently incorporated by the authors using Microsoft PowerPoint. ChatGPT was not used to determine, calculate, or validate the temperature ranges presented in the figure. The final illustration was reviewed and edited by the authors before its inclusion in the manuscript.

## 3. Results and Discussion

### 3.1. Thermal Characterization by DSC and TGA

**Figure 6:**
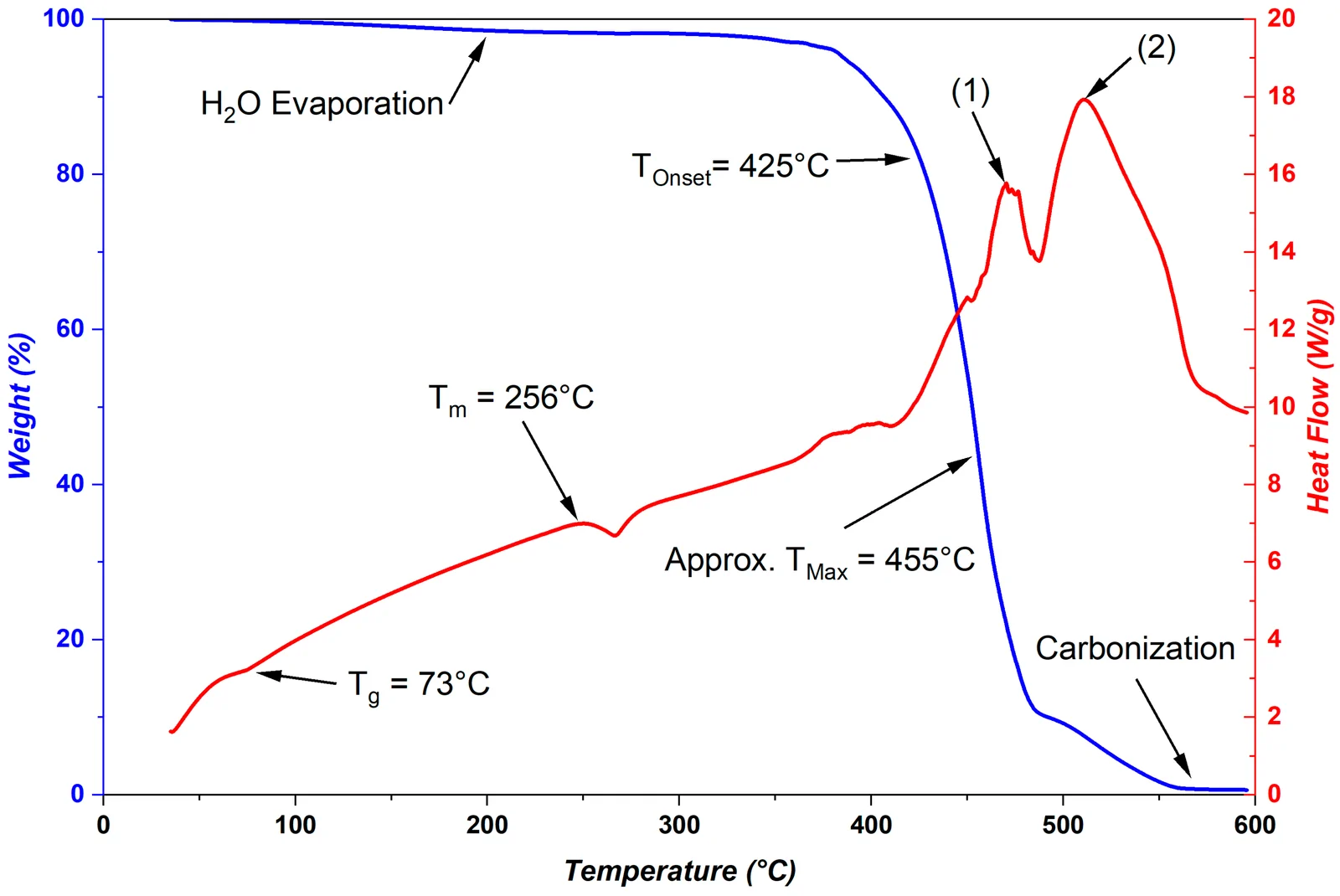
Simultaneous thermal analysis (SDT Q600) thermogram of PA66 IM HS showing the heat-flow and thermogravimetric signals, including the melting transition (~256 °C), multi-stage thermal degradation with onset at ~425 °C and maximum degradation near ~455 °C, and two heat-flow peaks within the degradation region at approximately (1) 470 °C and (2) 513 °C.

Figure 6 presents the combined TGA–DSC thermogram of PA66 IM HS, illustrating both the thermal stability and degradation behavior of the material. The TGA curve shows that the material maintains a nearly constant mass up to approximately 420 °C, indicating high thermal stability within the temperature range relevant to the mechanical testing (up to 150 °C). A significant weight loss is observed beyond this temperature, with the onset of degradation occurring around 420–450 °C, followed by a major decomposition stage between approximately 450 °C and 520 °C. This behavior is associated with the breakdown of the polymer backbone and volatilization of degradation products. The negligible residual mass at higher temperatures confirms the predominantly organic nature of the material.

As shown in Figure 6, the heat-flow signal obtained from the SDT Q600 analysis indicates a thermal transition at approximately 73 °C, which is assigned to the glass-transition region of PA66 IM HS. The experimental tensile results show a pronounced reduction in stiffness and strength between the tests conducted at 23 °C and 85 °C, accompanied by an increase in ductility. Although this mechanical evolution occurs across the temperature interval containing the identified glass-transition region, the present results do not establish a direct mechanistic relationship between Tg and the observed tensile response. Confirmation of the temperature-dependent changes in viscoelastic properties would require complementary dynamic mechanical analysis (DMA). Furthermore, the heat-flow signal obtained from the SDT Q600 analysis showed a melting endotherm at approximately 256 °C, consistent with the melting transition of PA66 IM HS. This indicates that all mechanical tests conducted up to 150 °C remained well below the melting and thermal degradation regions. Therefore, the observed reductions in stiffness and strength are consistent with temperature-dependent softening of PA66 IM HS rather than thermal degradation under the investigated testing conditions.

**Figure 7:**
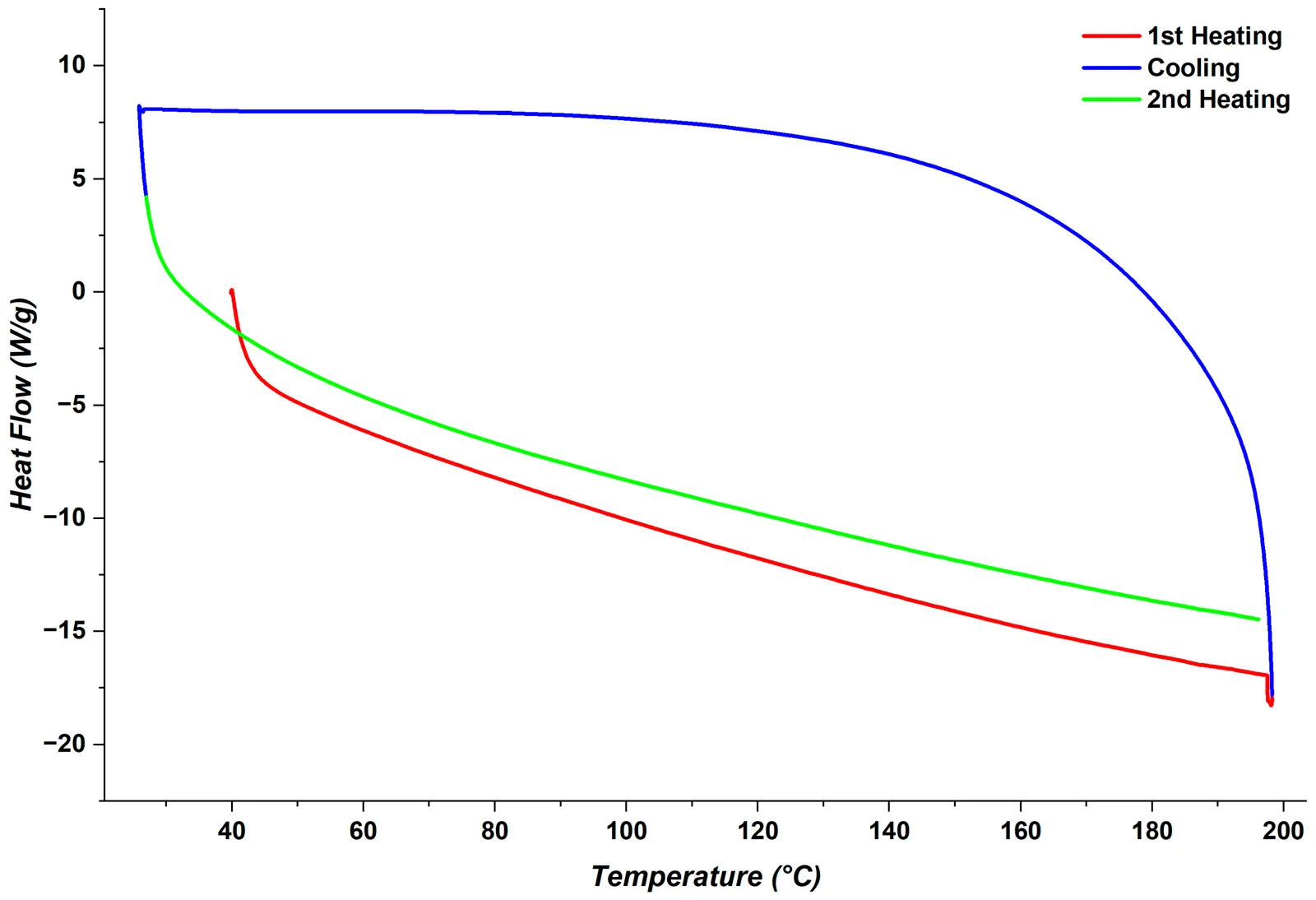
DSC thermograms of PA66 IM HS during the first heating and cooling and second heating cycles.

Figure 7 presents the DSC thermogram of PA66 IM HS, including the first heating, cooling, and second heating cycles. Differences among these thermal cycles may reflect the influence of the previous processing and thermal history of the material. However, because crystallization and melting enthalpies were not quantitatively determined, the effect of thermal history on the degree of crystallinity was not evaluated in the present study.

The TGA results therefore confirm that the mechanical tests conducted up to 150 °C were performed well below the onset of thermal decomposition of PA66 IM HS under the experimental conditions used in this study. However, these results should not be interpreted as evidence of long-term automotive durability or heat-aging resistance. The present TGA analysis does not reproduce prolonged thermo-oxidative exposure, hydrothermal aging, or other environmental conditions that may occur during automotive service. Evaluation of these effects would require dedicated long-term aging and environmental testing.

### 3.2. Experimental Stress–Strain Behavior

**Figure 8:**
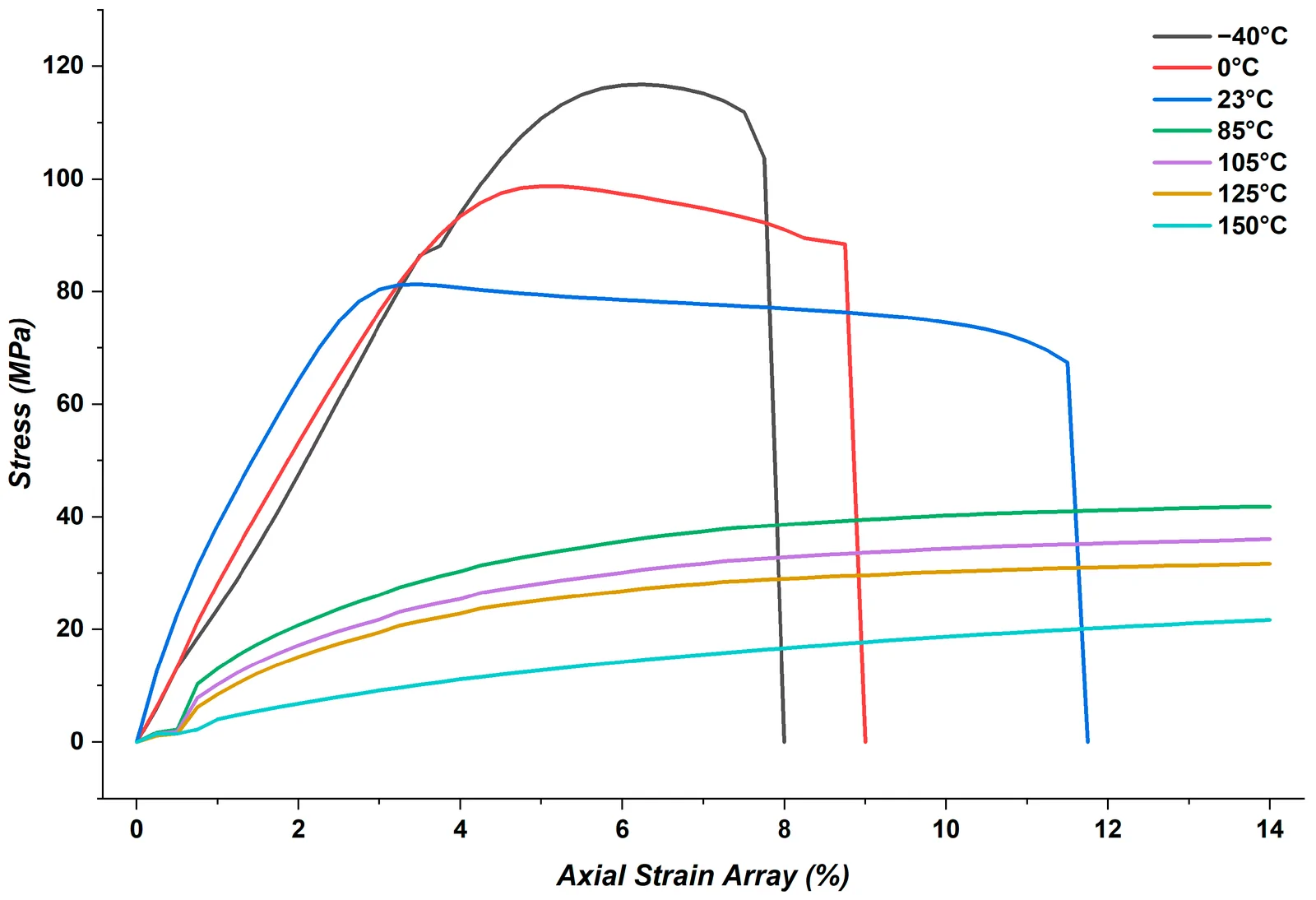
Experimental average stress–strain curves of PA66 IM HS at different testing temperatures.

Figure 8 shows the experimental stress–strain curves of PA66 IM HS evaluated at temperatures of −40 °C, 0 °C, 23 °C, 85 °C, 105 °C, 125 °C and 150 °C. The mechanical response exhibits a strong temperature dependence, characterized by a progressive reduction in yield strength, ultimate tensile strength, and elastic stiffness, accompanied by an increase in elongation at break as temperature increases. This behavior is consistent with the temperature-dependent softening commonly observed in polyamide materials and with the transition toward a more ductile mechanical response at elevated temperatures. At −40 °C, the material exhibits a predominantly brittle response, characterized by high strength and limited deformation before fracture. The ultimate tensile strength (UTS) reaches approximately 116.83 MPa at a strain of 6.25%, followed by a rapid stress decrease and fracture at approximately 7.75% strain. This behavior indicates limited post-UTS deformation under the low-temperature testing condition.

At 0 °C, the material maintains relatively high strength while exhibiting greater post-UTS deformation than at −40 °C. The UTS reaches approximately 98.72 MPa at 5.00% strain, followed by progressive deformation until fracture at approximately 8.75% strain.

At room temperature (23 °C), PA66 IM HS exhibits a more pronounced post-yield deformation response. The UTS reaches approximately 81.30 MPa at 3.50% strain. After reaching the maximum stress, the material undergoes progressive post-yield deformation, with the stress decreasing to approximately 67.45 MPa before fracture at approximately 11.50% strain. At elevated temperatures (85–150 °C), a significant decrease in stress level is observed, accompanied by extended deformation before failure. At 85 °C, the polymer exhibits a stable plastic deformation region after yielding, reaching a maximum stress of approximately 41.85 MPa. This trend becomes more pronounced at 105, 125, and 150 °C, where progressively lower stress levels and greater deformation are observed, indicating continued temperature-dependent softening of the material.

The highest testing temperatures (125 and 150 °C) show the lowest stress levels, with maximum stresses of approximately 31.6 MPa and 21.69 MPa, respectively. Nevertheless, the material maintains continuous deformation without abrupt failure, demonstrating the effectiveness of the impact-modified and heat-stabilized formulation in preserving mechanical integrity under elevated thermal conditions. Overall, the stress–strain evolution demonstrates a strong temperature dependence in the deformation behavior of PA66 IM HS. The decrease in stiffness and strength with increasing temperature is accompanied by enhanced ductility, which is relevant for automotive applications subjected to variable operating temperatures.

To facilitate the quantitative comparison of the temperature-dependent mechanical response, the main tensile properties extracted from the experimental stress–strain curves are summarized in Table 2. These parameters include the UTS, the corresponding strain at UTS, the fracture stress, and the fracture strain. For specimens tested at 85 °C and above, fracture was not observed within the maximum applied strain (14%), and therefore the reported values correspond to the highest stress reached during the test.

### 3.3. Model Prediction vs. Experimental Data

**Figure 9:**
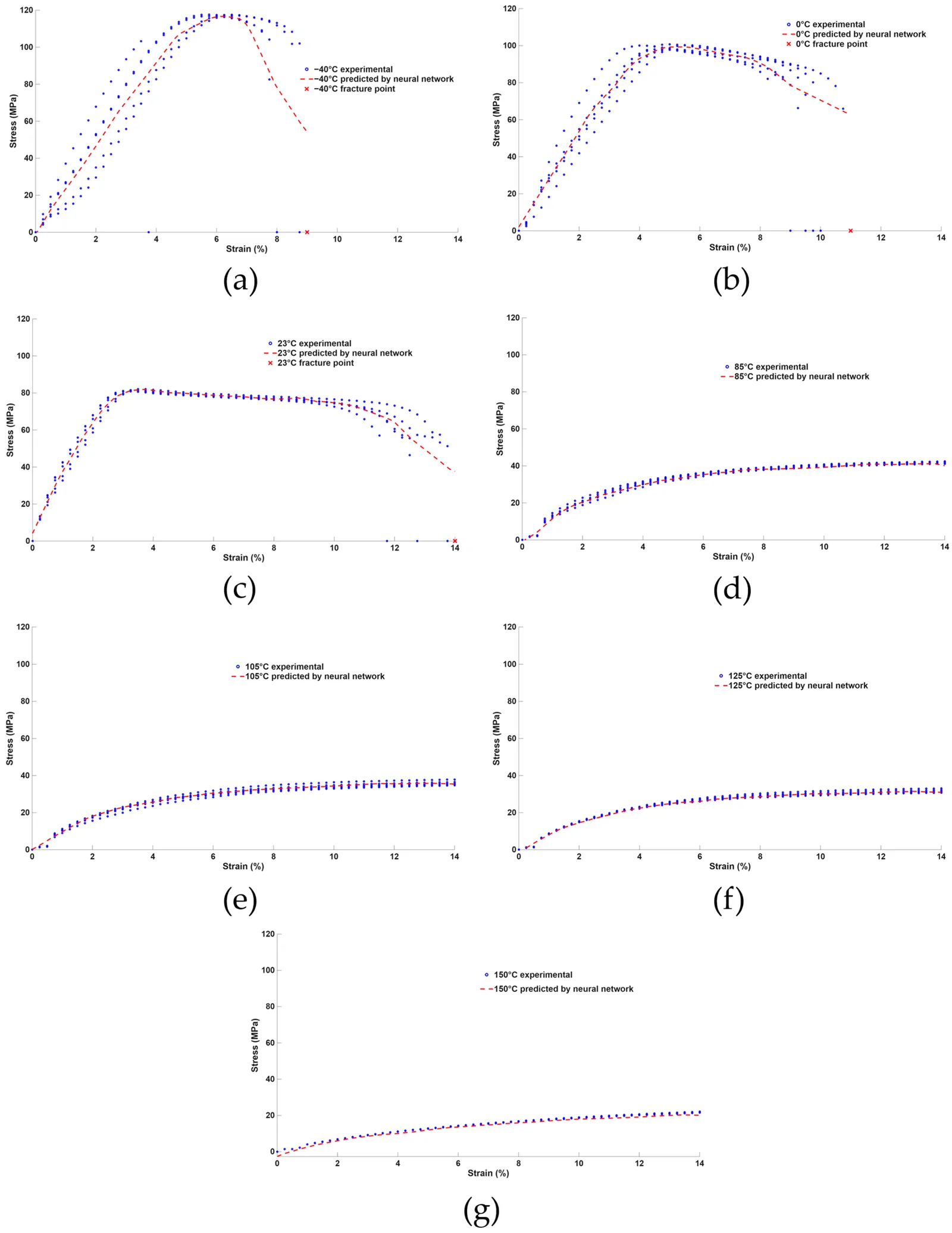
Comparison between experimental samples stress–strain curves and model prediction at (**a**) −40 °C, (**b**) 0 °C, (**c**) 23 °C, (**d**) 85 °C, (**e**) 105 °C, (**f**) 125 °C, (**g**) 150 °C.

Figure 9 compares the stress–strain curves predicted by the proposed DNN with the five experimental tensile tests carried out at each assessed temperature. Overall, the predicted curves track the mean experimental response across the full temperature range while staying within the envelope defined by the experimental specimens. The magnitude of the experimental scatter depends on temperature. The greatest dispersion occurs at −40 °C and 0 °C, especially after the UTS and in the vicinity of the fracture region, whereas the curves obtained at 85–150 °C show substantially less specimen-to-specimen variability throughout the entire deformation process.

The higher variability observed at sub-zero temperatures is associated with the brittle fracture behavior of PA66 IM HS. Under these conditions, fracture occurs within a comparatively limited deformation range, making the measured fracture strain more sensitive to specimen-to-specimen experimental variability. Conversely, at elevated temperatures, the greater deformation capacity of the material results in smoother and more reproducible stress–strain responses across the experimental specimens.

The proposed DNN determines the full stress–strain path by learning the nonlinear mapping among strain, temperature, and stress from the experimental database. In the training stage, the network repeatedly adjusts the weights and biases of its three fully connected hidden layers via backpropagation with the Adam optimization algorithm, thereby reducing the prediction error between the computed and experimental responses. As a result, the proposed DNN learns the nonlinear relationship between strain, temperature, and stress from the experimental dataset, enabling smooth interpolation of tensile stress–strain responses within the investigated temperature domain.

The predicted curves closely reproduce the elastic region, the yielding transition, and the post-yield progression across the experimentally characterized temperatures. The correspondence is especially strong at 85 °C, 105 °C, 125 °C, and 150 °C, where the predicted curves closely coincide with the mean experimental response. Minor discrepancies are observed primarily near the fracture region at −40 °C and 0 °C, where the stochastic character of brittle fracture increases the variability among experiments. Even so, the predicted curves remain within the experimental scatter band, indicating that the proposed approach captures the main temperature-dependent tensile response of PA66 IM HS under the investigated testing conditions rather than reproducing individual experimental curves. Overall, these findings indicate that the DNN reproduces the main temperature-dependent tensile trends represented in the experimental dataset under the investigated testing conditions.

### 3.4. Error Analysis

**Figure 10:**
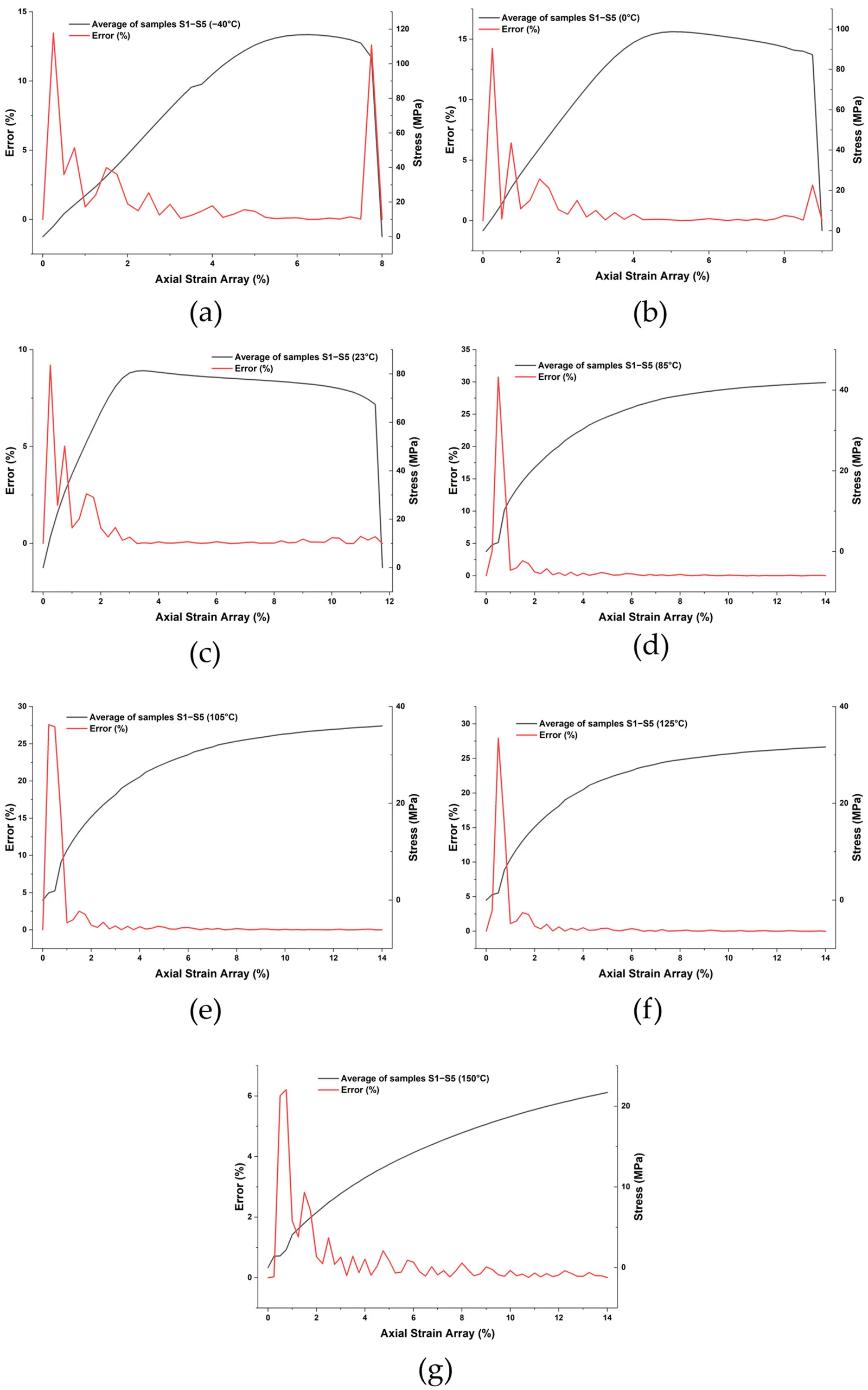
Error percentage vs. average strain at: (**a**) −40 °C, (**b**) 0 °C, (**c**) 23 °C, (**d**) 85 °C, (**e**) 105 °C, (**f**) 125 °C, (**g**) 150 °C.

The agreement between the experimental and predicted stress values was evaluated by calculating the percentage error between the experimental and predicted stress values. The error was determined using the relative difference between both datasets across the strain range. Figure 10 shows the distribution of the prediction error as a function of strain. The results indicate that the error remains relatively low throughout most of the deformation range, with slightly higher deviations observed at larger strain values.

In both scenarios, the most pronounced error peaks are concentrated within the initial strain domain, specifically within an axial strain range of approximately 0% to 1.8%. This phenomenon is attributed to the initial setting of the specimen and the load stabilization phase during tensile assessments, wherein both the material and the gripping mechanism gradually adapt to the imposed tensile load. Within this phase, minor misalignments, grip settling, micro-slippage, and the progressive elimination of mechanical slack can result in transient variations in the recorded stress values.

Consequently, the empirical data within the nascent deformation region exhibit heightened variability, culminating in localized spikes in the computed prediction error. Once the specimen attains a stable tensile condition and the material is fully engaged under load, the error diminishes rapidly and remains low throughout most of the deformation range. This behavior indicates closer agreement between the predicted and experimental responses after the initial stabilization phase. The localized error peaks observed at the onset of testing may therefore reflect, at least in part, the sensitivity of the experimental measurements during the initial loading phase. The near-zero error sustained across the intermediate deformation range further indicates good agreement between the predicted and experimental responses under the testing conditions represented in the dataset.

### 3.5. LOTO Validation of the DNN Model

To assess the interpolation capability of the DNN beyond the conventional random train–validation–test partition, a leave-one-temperature-out (LOTO) validation was performed using the five interior experimental temperatures. In each case, all experimental data corresponding to one temperature were completely withheld from model development and subsequently used as an independent experimental reference for evaluating the predicted stress–strain response. This approach provides a more stringent assessment of temperature interpolation because the model is required to estimate the mechanical response at a thermal condition not represented during its development, while experimental data remain available for direct comparison. Table 3 summarizes the predictive performance obtained for the five withheld temperatures in terms of RMSE, MAE, and R^2^.

**Figure 11:**
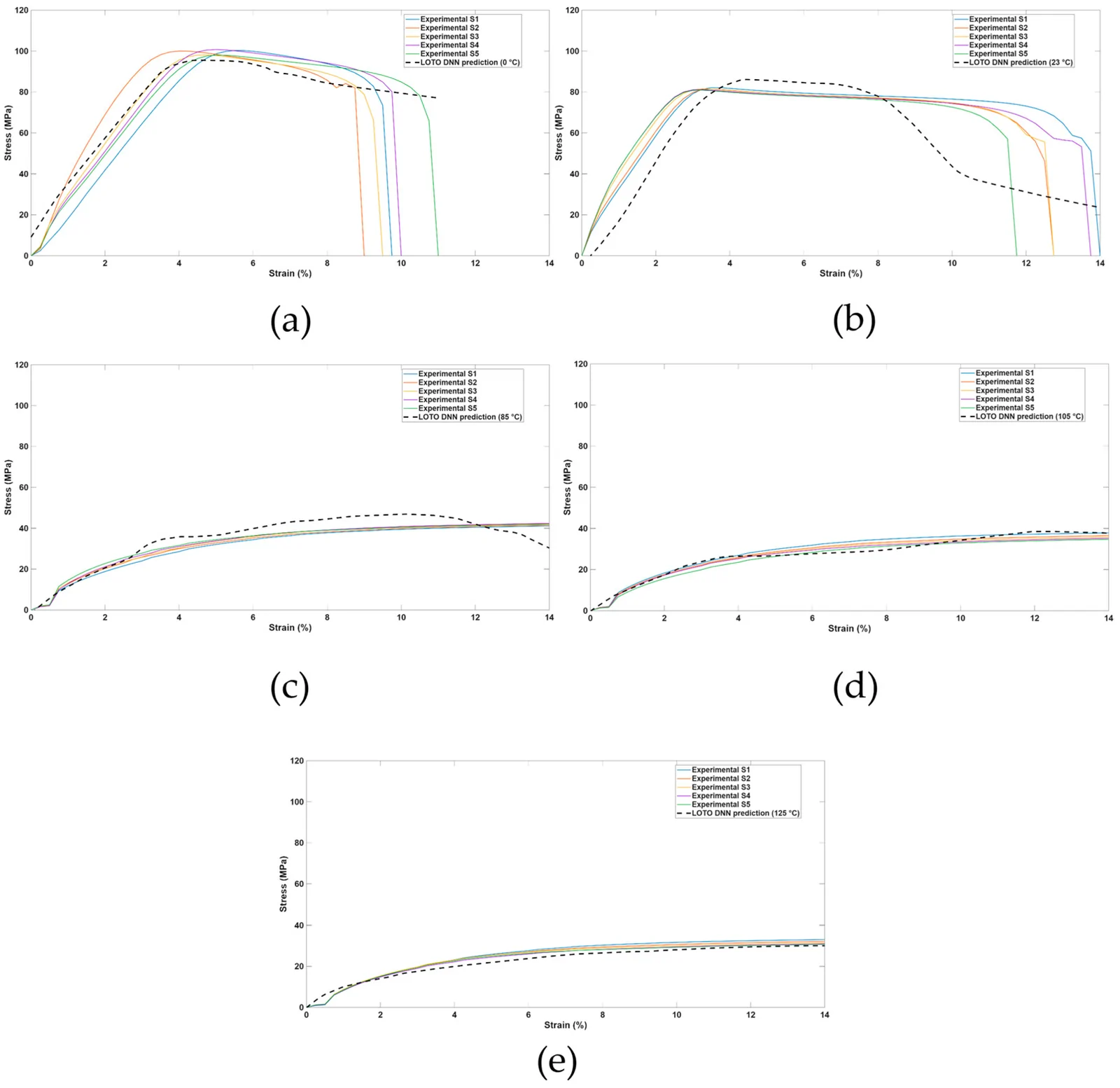
LOTO validation results for (**a**) 0 °C, (**b**) 23 °C, (**c**) 85 °C, (**d**) 105 °C, (**e**) 125 °C.

The LOTO results revealed a clear dependence of prediction accuracy on the withheld thermal condition. The highest predictive performance was obtained at 105 °C and 125 °C, with RMSE values of 2.346 and 2.401 MPa, MAE values of 1.935 and 2.154 MPa, and R^2^ values of 0.940 and 0.920, respectively. At 85 °C, the model maintained reasonable agreement with the withheld experimental data, although the prediction error increased to an RMSE of 4.922 MPa and an MAE of 4.299 MPa, with an R^2^ of 0.798. In contrast, substantially larger deviations were observed when 0 °C and 23 °C were excluded, resulting in RMSE values of 14.612 and 19.740 MPa, respectively. The 23 °C condition represented the most challenging holdout case, with an MAE of 15.624 MPa and an R^2^ of 0.038. These results demonstrate that the interpolation accuracy of the reduced-input DNN is not uniform throughout the investigated temperature domain and depends strongly on the thermal region represented by the available experimental training data. Figure 11 presents the corresponding LOTO stress–strain predictions together with the five experimental curves for each withheld temperature, allowing the differences in predictive performance across the investigated thermal conditions to be visualized directly.

The LOTO predictions reproduced the experimental stress–strain behavior more closely in the elevated-temperature region, particularly at 105 °C and 125 °C, where the predicted curves remained close to the experimental responses throughout most of the deformation range. The 85 °C prediction captured the overall shape of the experimental response but showed increasing deviations at intermediate and higher strain levels. Greater discrepancies were observed for the low-temperature holdout conditions. At 0 °C, the DNN reproduced the general stress–strain trend but did not capture the experimentally observed terminal fracture behavior because fracture information from the withheld condition was intentionally excluded from the LOTO procedure. The largest discrepancy occurred at 23 °C, where deviations were observed over a substantial portion of the stress–strain curve rather than being restricted to the fracture region. When 23 °C was withheld, the nearest available experimental temperatures were 0 °C and 85 °C, leaving a comparatively wide temperature interval in which the mechanical response changes substantially. The LOTO results therefore indicate that prediction accuracy is sensitive to the distribution and spacing of the available experimental temperatures, particularly in regions where the mechanical response varies markedly with temperature. Consequently, the predictions generated at 10 °C, 95 °C, and 140 °C should be interpreted as interpolation estimates within the investigated thermal domain rather than as independently validated experimental predictions.

### 3.6. Intermediate-Temperature Prediction

**Figure 12:**
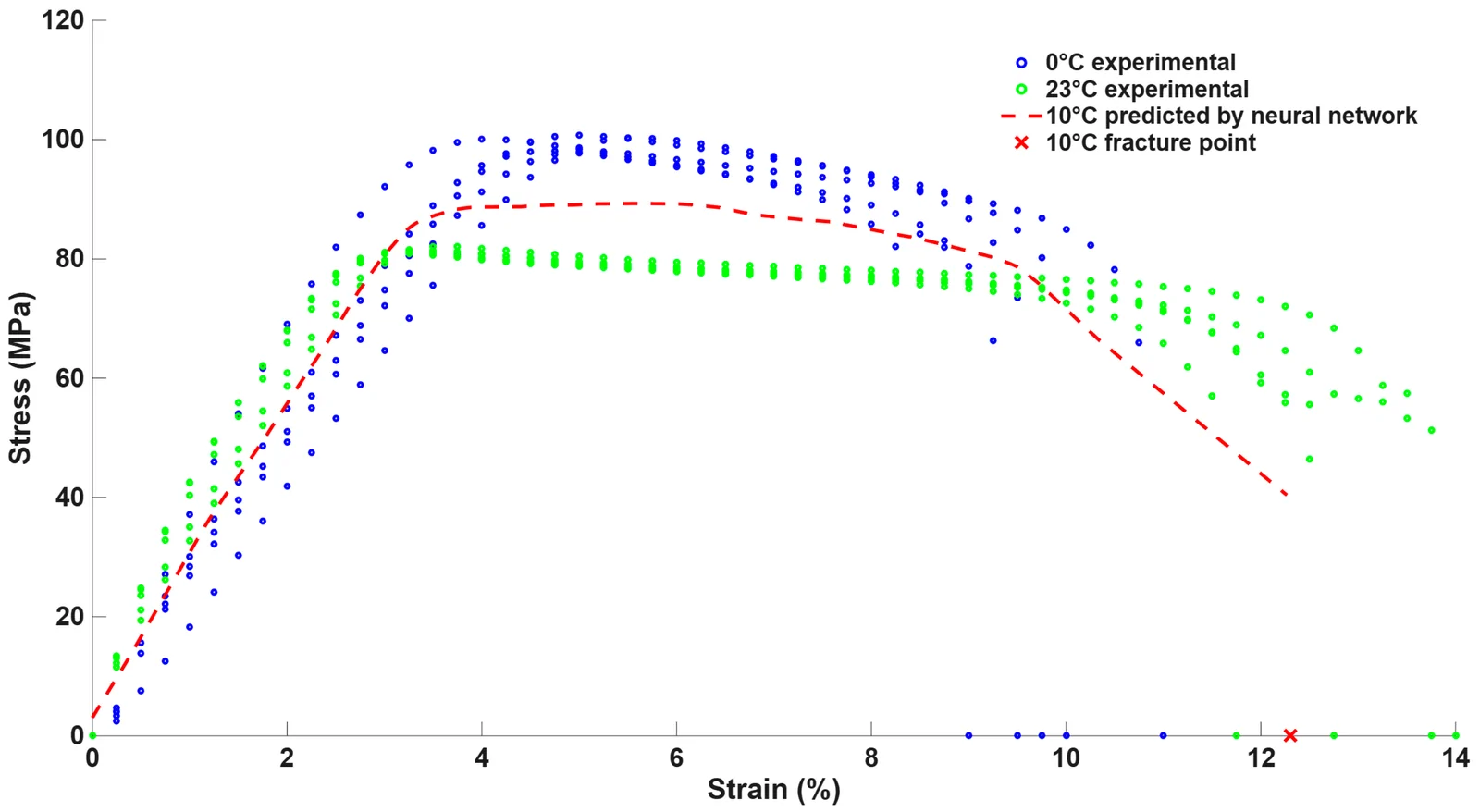
DNN-predicted stress–strain curve at 10 °C.

**Figure 13:**
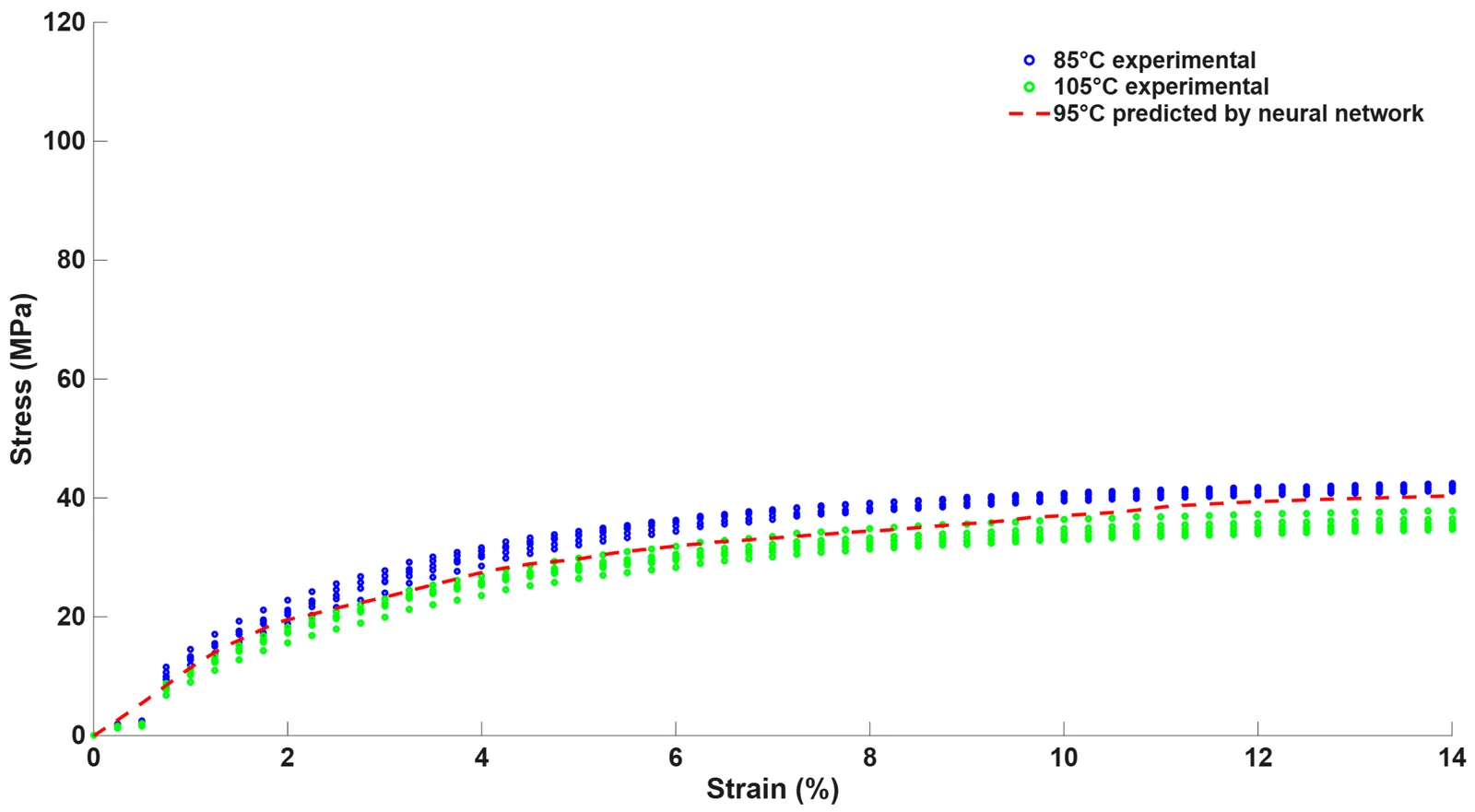
DNN-predicted stress–strain curve at 95 °C.

**Figure 14:**
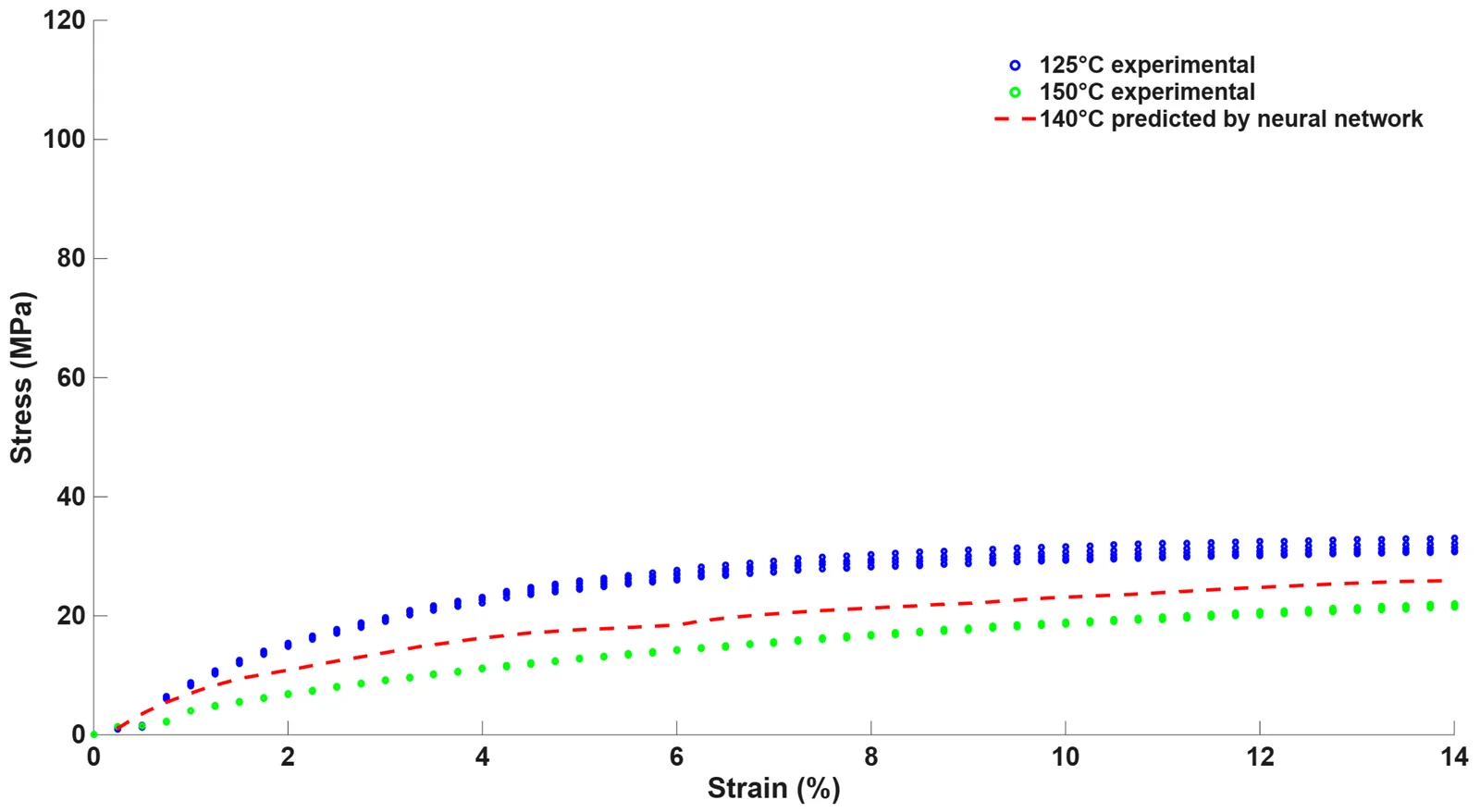
DNN-predicted stress–strain curve at 140 °C.

To evaluate the interpolation capability of the proposed methodology, the model was used to estimate the stress–strain behavior of PA66 IM HS at intermediate temperatures not included in the experimental training dataset. DNN-predicted stress–strain curves were generated at 10 °C (Figure 12), 95 °C (Figure 13) and 140 °C (Figure 14).

The predicted stress–strain curves exhibited physically consistent temperature-dependent tensile behavior, with the mechanical properties falling within the ranges defined by the neighboring experimental temperatures. For the predicted response at 10 °C, the model estimated an ultimate tensile stress of 89.35 MPa occurring at 5.34% strain, which is bound by the experimental values obtained at 0 °C (98.72 MPa) and 23 °C (81.30 MPa). Likewise, the predicted curves at 95 °C and 140 °C reached maximum stresses of 39.22 MPa and 26.06 MPa, respectively, remaining between the corresponding neighboring experimental temperatures (85–105 °C and 125–150 °C).

In addition to preserving the magnitude of the mechanical properties, the proposed model consistently reproduced the characteristic evolution of the stress–strain response with increasing temperature, including the reduction in elastic stiffness, the gradual decrease in ultimate tensile strength, and the increase in ductility. The predicted curves exhibited smooth transitions without discontinuities or numerical oscillations, indicating that the DNN generated continuous interpolated responses across the investigated temperature domain.

These results indicate that the proposed methodology provides physically consistent interpolated responses within the experimentally characterized temperature range. The generated stress–strain responses may provide complementary data for applications such as finite element material calibration and temperature-dependent material modeling; however, experimental validation remains necessary when extending the model to previously uncharacterized testing conditions.

### 3.7. Comparison with Other Works

Most of the methods reported in the literature for predicting the mechanical response of materials using machine-learning approaches employ artificial neural networks, deep neural networks, or recurrent architectures to predict nonlinear stress–strain behavior (Table 4). These approaches have demonstrated their ability to capture complex relationships between material parameters and mechanical response that are difficult to represent using conventional constitutive models alone. However, several reported studies have been developed at room temperature or within relatively narrow temperature ranges, limiting their applicability when strong temperature-dependent behavior must be considered. For example, Saha et al. applied a deep neural network to predict microscale elastoplastic stress and strain fields in a two-phase material, whereas Savall et al. employed a Long Short-Term Memory architecture to generate stress–strain curves based on established constitutive material laws. Other studies have incorporated temperature as an input variable. Teng et al., for instance, used artificial neural networks to model the mechanical behavior of PET within temperature ranges of approximately 85–110 °C and 95–115 °C, accounting for the influence of temperature, strain and strain rate. Similarly, Mao et al. developed a deep-learning framework for predicting stress–strain curves of Ni-based superalloys, although the reported analysis was focused on room-temperature conditions.

The present study demonstrated that a DNN can reproduce the temperature-dependent stress–strain behavior of injection-molded PA66 IM HS over a wide temperature range from −40 °C to 150 °C using experimentally obtained tensile data. The proposed methodology successfully reproduced the elastic region, yielding, post-yield deformation, and low-temperature fracture behavior, achieving strong agreement with the experimental stress–strain curves across all evaluated temperatures. Thermal characterization confirmed that PA66 IM HS remained thermally stable throughout the mechanical testing range. The glass-transition region was identified at approximately 73 °C, while the heat-flow signal obtained from the SDT Q600 analysis showed a melting transition near 256 °C. TGA revealed the onset of thermal degradation above 425 °C. These findings indicate that the variations observed in the tensile response occurred well below the thermal degradation region and are therefore consistent with temperature-dependent mechanical softening rather than degradation of the polymer.

The experimental tensile characterization revealed a pronounced dependence of the mechanical response on temperature. The UTS decreased from 116.83 MPa at −40 °C to 21.69 MPa at 150 °C, representing an approximate 81% reduction, while the fracture strain increased from 7.75% at −40 °C to values exceeding 14% at elevated temperatures. These results demonstrate a progressive transition from brittle to ductile behavior with increasing temperature, highlighting the strong influence of thermal conditions on the mechanical performance of PA66 IM HS. The proposed DNN generated physically consistent interpolated stress–strain responses at 10 °C, 95 °C, and 140 °C. The predicted ultimate tensile strengths of 89.35 MPa, 39.22 MPa, and 26.06 MPa, respectively, remained within the mechanical envelopes defined by the neighboring experimental datasets. These results indicate that the model preserves the experimentally observed temperature-dependent tensile trends within the investigated thermal domain; however, because experimental tensile data were not available at these intermediate temperatures, these predictions should be interpreted as interpolation estimates rather than independent experimental validation. Furthermore, the incorporation of the proposed fracture-aware prediction strategy prevented stress predictions beyond the experimentally observed fracture strain under the low-temperature brittle conditions considered in this study, thereby improving the physical consistency of the generated curves.

Overall, the proposed methodology provides a data-driven framework for estimating the temperature-dependent monotonic tensile stress–strain behavior of PA66 IM HS within the experimentally characterized thermal domain. By combining experimental tensile characterization, DNN learning, and fracture-aware post-processing, the framework generates physically consistent interpolated stress–strain responses that may support applications such as material modeling and finite element calibration. The present model is restricted to the experimental conditions represented in the dataset and does not explicitly account for strain-rate dependence, moisture effects, time-dependent viscoelastic behavior, or loading history. Extension of the methodology to other loading conditions, environmental states, or engineering thermoplastics would therefore require additional experimental data and independent validation.

## 4. Conclusions

The proposed DNN successfully reproduced the main temperature-dependent trends of the tensile stress–strain behavior of injection-molded PA66 IM HS over the investigated temperature range (−40 °C to 150 °C), capturing the elastic, yielding, post-yield, and low-temperature fracture regimes represented in the experimental dataset. PA66 IM HS exhibited a strong temperature-dependent mechanical response. The UTS decreased from 116.83 MPa at −40 °C to 21.69 MPa at 150 °C, corresponding to an approximately 81% reduction, while fracture strain increased from 7.75% to values above 14%, indicating a progressive transition toward more ductile tensile behavior with increasing temperature.

The heat-flow signal obtained from the SDT Q600 analysis identified a glass-transition region at approximately 73 °C and a melting transition near 256 °C, while the thermogravimetric signal indicated the onset of thermal degradation at approximately 425 °C. The proposed DNN framework reproduced the experimentally characterized tensile responses and provided stress–strain interpolation estimates at intermediate temperatures within the investigated thermal domain. Predictions at 10 °C, 95 °C, and 140 °C followed the temperature-dependent mechanical trends established by the experimental dataset, yielding UTS values of 89.35 MPa, 39.22 MPa, and 26.06 MPa, respectively. These predictions should be interpreted as interpolation estimates rather than independent experimental validations. In addition, the fracture-aware prediction strategy prevented non-physical continuation of predicted stress beyond the estimated fracture strain within the experimentally supported low-temperature range.

The leave-one-temperature-out validation provided an independent assessment of the interpolation capability of the DNN and demonstrated that predictive accuracy was not uniform throughout the investigated thermal domain. The strongest agreement with withheld experimental data was obtained at 105 °C and 125 °C, with RMSE values of 2.346 and 2.401 MPa and R^2^ values of 0.940 and 0.920, respectively. At 85 °C, the model yielded an RMSE of 4.922 MPa and an R^2^ of 0.798. Larger deviations were observed when 0 °C and 23 °C were withheld, with RMSE values of 14.612 and 19.740 MPa, respectively, and the 23 °C condition produced the lowest agreement (R^2^ = 0.038). These results indicate that the interpolation performance of the reduced-input DNN depends on the distribution of the available experimental temperatures and on the magnitude of the mechanical-response changes between neighboring thermal conditions.

Overall, the results demonstrate the potential of the proposed methodology as a computational tool for estimating temperature-dependent monotonic tensile behavior within the experimentally characterized thermal domain. The framework can provide complementary stress–strain estimates between experimentally characterized temperatures and support material evaluation and engineering modeling activities; however, the LOTO results demonstrate that experimental coverage remains important, particularly in thermal regions exhibiting pronounced changes in mechanical response. Accordingly, predictions at previously uncharacterized temperatures should not be considered substitutes for experimental validation, and additional experimental data may be required when higher predictive accuracy is needed or when the available training temperatures are widely spaced.

## Figures and Tables

**Table 1:** PA66 IM HS—material specification [53].

Property	Units	Test Method	Limits
Melting Point	°C	ASTM D3418	255–267
Density	g/cm^3^	ASTM D792	1.10–1.15
Moisture Content	% max	ASTM D789	0.22
Tensile Stress at Yield	MPa min	ASTM D638	65–68
Tensile Strain at Break	% min	ASTM D638	15–30

**Table 2:** Mechanical properties extracted from the experimental stress–strain curves of PA66 IM HS at different testing temperatures.

Temperature (°C)	Ultimate Tensile Strength (MPa)	Strain at UTS (%)	Fracture Stress (MPa)	Strain at Fracture (%)
−40	116.83	6.25	103.69	7.75
0	98.72	5	88.47	8.75
23	81.3	3.5	67.45	11.50
85	41.85	14	41.85	No fracture reached
105	35.99	14	35.99	No fracture reached
125	31.67	14	31.67	No fracture reached
150	21.69	14	21.69	No fracture reached

**Table 3:** LOTO validation results.

Excluded Temperature (°C)	LOTO RMSE (MPa)	LOTO MAE (MPa)	LOTO R^2^
0	14.612	8.214	0.78
23	19.74	15.624	0.038
85	4.922	4.299	0.798
105	2.346	1.935	0.94
125	2.401	2.154	0.92

**Table 4:** Comparison of machine-learning approaches for predicting mechanical behavior across different temperature ranges.

Authors	Method	Temperature Range (°C)	Observations	Reference
Soto et al.	Deep Neural Network	−40 to 150	A deep neural network was used to estimate the temperature-dependent stress–strain behavior of injection-molded PA66 IM HS from −40 to 150 °C. The model reproduced the nonlinear mechanical response and fracture behavior at experimentally characterized temperatures and generated physically consistent interpolated responses at intermediate temperatures within the investigated thermal domain.	This work
Saha et al.	Deep Neural Network	Room temperature	The paper presents a deep learning framework that predicts micro-scale elasto-plastic strains and stresses in a two-phase medium, addressing the challenge of inelastic behavior in materials.	[54]
Savall et al.	Long Short-Term Memory	Room temperature	It generates stress–strain curves based on specific material parameters using established material laws, namely the von Mises material law and the Ramberg-Osgood equation.	[55]
Fei Teng et al.	Artificial Neural Network	85 to 110	It addresses the complexities of modeling PET behavior, which is influenced by multiple parameters such as temperature, strain, and strain rate.	[56]
	Artificial Neural Network	95 to 115	The model combines a physics-based function for small strain behavior with an ANN model for temperature-dependent large strain nonlinear viscoelastic behavior.	[57]
Yuwei Mao et al.	Deep Learning Framework	Room temperature	The model focuses specifically on predicting stress–strain curves for Nickel (Ni)-based superalloys, relevant to the aerospace industry.	[58]

## Data Availability

The original contributions presented in this study are included in the article. Further inquiries can be directed to the corresponding author.
